# Low-power laser manufacturing of copper tracks on 3D printed geometry using liquid polyimide coating†

**DOI:** 10.1039/d3na00120b

**Published:** 2023-03-18

**Authors:** Mansour Abdulrhman, Adarsh Kaniyoor, Carmen M. Fernández-Posada, Pablo Acosta-Mora, Ian McLean, Nick Weston, Marc P. Y. Desmulliez, Jose Marques-Hueso

**Affiliations:** a School of Engineering and Physical Sciences, Institute of Sensors, Signals and Systems, Heriot-Watt University EH14 4AS Edinburgh UK J.Marques@hw.ac.uk; b Department of Physics, Maxwell Centre J. J. Thomson Avenue CB3 0HE Cambridge UK; c Renishaw plc. Research Avenue, Riccarton Edinburgh EH14 4AP UK

## Abstract

Silver nanoparticle photoreduction synthesis by direct laser writing is a process that enables copper micro-track production on very specific polymers. However, some important 3D printing polymers, such as acrylonitrile butadiene styrene (ABS) and acrylates, do not accept this treatment on their surface. This work presents an approach to produce copper microcircuitry on 3D substrates from these materials by using direct laser writing at low power (32 mW CW diode laser). We show that by coating a thin layer of polyimide (PI) on a 3D-printed geometry, followed by a sequence of chemical treatments and low-power laser-induced photoreduction, copper tracks can be produced using silver as catalyst. The surface chemistry of the layer through the different stages of the process is monitored by FTIR and X-ray photoelectron spectroscopy. The copper tracks are selectively grown on the laser-patterned areas by electroless copper deposition, with conductivity (1.2 ± 0.7) × 10^7^ S m^−1^ and a width as small as 28 μm. The patterns can be written on 3D structures and even inside cavities. The technique is demonstrated by integrating different circuits, including a LED circuit on 3D printed photopolymer acrylate and a perovskite solar cell on an ABS 3D curved geometry.

## Abstract 2

A low-power laser patterning method enables the production of copper microcracks on 3D surfaces of some important 3D printing polymers, such as ABS and acrylate photopolymer. Tracks as small as 28 μm are produced with a simple 32 mW CV laser diode.

## Graphical abstract

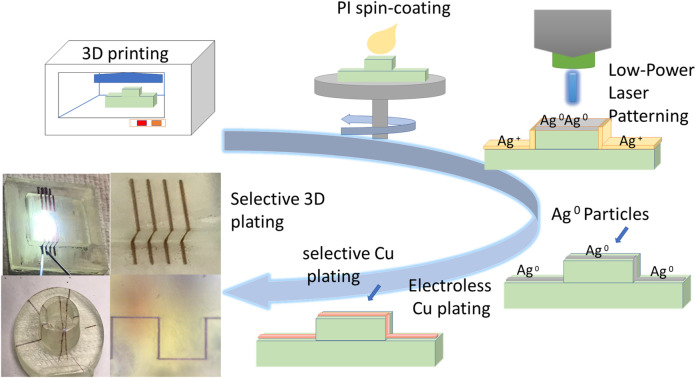

## 1. Introduction

Selective and controlled plating of a conductive circuit on different polymeric substrates is of great interest in many research fields, especially for microelectronic applications for the interconnection of electronic devices, micro sensors, wearable devices, *etc.* Various techniques have been used to deposit metal contacts on polymer substrates. Methods like inkjet printing^1,2^ chemical vapour deposition^3,4^ and electroless deposition^5–7^ are currently the most widely used techniques, particularly in industrial mass production. Laser-based processes usually rely on the use of high-power lasers for depositing metals such as copper on polymeric materials and working in the ablation regime. Some examples include laser direct structuring (LDS),^8–11^ femtosecond laser reductive sintering and laser-induced forward transfer (LIFT).^12–14^ A distinct advantage of using lasers for photopatterning is the ability to modify substrates without applying any mechanical contact.^15^ Further, the ability to control a laser wavelength and power can help to achieve precise control of penetration depth and track shape (and width), which is required for specific microcircuitry fabrication.^16^ These features allow for the simplistic and smooth integration of lasers into other fabrication devices such as printers, milling machines, *etc.* Lasers are both a source of illumination and heat, localised photochemical and photothermal reactions may be performed on substrates without damaging adjacent surfaces. Thus, lasers can become efficient instruments for patterning thermally sensitive substrates or flexible polymeric electrodes.^17^ However, the current laser-based methods, such as those mentioned above, use high-power and expensive equipment.

An alternative approach has been the development of solution-assisted laser patterning for writing circuits on polymeric substrates.^5,18,19^ Previously reported work successfully demonstrated the deposition of micro copper tracks on polyetherimide using optically reduced nanoparticles of Ag as a seed layer.^20,21^ In this case, photoreduction was achieved using a low-power visible light laser under ambient conditions. In this process, metal ions are used as the seed layer, with silver having shown a particularly high performance.^22,23^ Moreover, the combination of Ag^+^ ions (originally from the AgNO_3_ treatment) with dissolved KCl aids the formation of AgCl, which enables the Ag fastest photoreduction on the substrate, speeding up the writing process. This method is valid for materials that can provide an anchoring site for the seeds after hydrolysis, such as polyetherimide,^19^ polydimethylsiloxane (PDMS),^24^ and the biodegradable polymer polycaprolactone (PCL).^25^ A challenging limitation is to extend the process to any material, and even more so in 3D. Some useful 3D printing materials such as acrylates/methacrylates won't plate selectively unless modified before the manufacturing process.^26^ Following the encouraging results with low-power laser patterning, this paper demonstrates a new approach to produce copper patterns on simple 3D structures including 3D cavities.

This work demonstrates the rapid and high-resolution writing of 3D micro-electrical circuits by first forming a polyimide layer on a 3D printed geometry followed by direct laser writing of Ag nanoparticles and electroless copper plating. Ag nanoparticles are selectively formed on the substrates when a focused 405 nm wavelength laser is used, therewith providing a seed layer for the selective deposition of copper on a polymer substrate. FT-IR and XPS characterizations are carried out during the various stages of the process to observe the modification of the substrate surface and understand its chemistry. Conductivity of the copper tracks are in the range of bulk copper, as determined using two-point probe measurements. Finally, as a proof-of-concept, the technique is used to integrate a solar cell into a curved geometry. The technique has been demonstrated to work on two different polymers (acrylic photopolymer and ABS), and given that it uses a polyimide coating, it is reasonable to expect that it would work with any material that provides enough adhesion to the polyimide.

## 2. Experimental

### 2.1. Materials

All the chemicals were used as received without further purification. PI-1388 Liquid Polyimide was purchased from VTEC™, iso-propanol and copper sulphate (CuSO_4_; 98%) were purchased from Sigma-Aldrich. Potassium hydroxide (KOH), silver nitrate (AgNO_3_), potassium tartrate (99%), ammonia (NH_3_; 18%) and sulfuric acid (H_2_SO_4_) were purchased from Fisher Scientific, UK. Sodium hydroxide (NaOH; 98.5%) and formaldehyde (37%) were purchased from Acros Organics.

### 2.2. Direct laser writing of 3D circuits

**Fig. 1:**
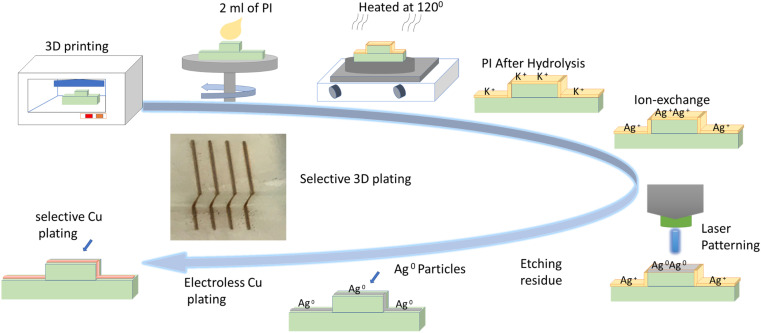
Flow chart illustrating the steps for depositing 3D laser-written micro-circuitry on a 3D printed sample *via* PI coating.

To test 3D geometries, a set of four 3D samples were prepared: two samples sized 1 × 2 mm for 2D Direct Laser Writing (DLW) cylindrical geometry with 4 mm outer diameter, and a 3D cubic structure (2 × 2 cm). All of these samples were 3D printed on an LCD 3D printer from Anycubic using a commercially available resin from Photocentric Ltd. 2 ml of liquid polyimide PI-1388 (PI) was spin-coated on the 3D printed substrates at 3000 rpm for 30 s, which were then heated at 120 °C for 20 minutes. The average thickness of the PI layer was approximately 230 μm, as measured by profilometry. Seeding of Ag nanoparticles (NPs) on PI coated substrates was achieved by a two-step reduction method reported elsewhere.^22^ Briefly, the substrates were hydrolysed by immersion in 15 M KOH solution for 20 min at 50 °C, cleaned with flowing DI water for 2 min (per side) and immersed in 0.1 M AgNO_3_ solution for 20 min to achieve ion-exchange. The substrates were sensitized by quick immersion (30 s) in 0.01 M KCl solution followed by drying at room temperature. The AgCl formed on the surface was photo-reduced by the direct laser writing method which uses a custom jig consisting of a low-power laser (100 mW, 405 nm, Violet Laser Module Dot Pattern model, from Odic Force Lasers, UK) fixed to a 5-axis CNC machine (Pocket NC V2-10, USA). The laser is an inexpensive common continuous-wave diode laser. The laser beam is focussed onto the substrate (power: 32 mW, and spot-diameter: 30 ± 2 μm) and moved programmatically to produce the required pattern. Ag tracks are formed on substrate regions which are exposed to the laser beam. Unexposed regions retain unexposed AgCl which was removed by immersion in 18% ammonia for 1 minute and cleaning with DI water for 2 minutes per side. This was followed by immersion in 5% sulfuric acid for 1 minute and rinsing with DI water for 2 minutes per side. Finally, the samples were immersed in an electroless copper plating bath at 50 °C for 5 minutes to achieve plating of Cu on Ag lines. A proof-of-concept working 3D electric circuit was also developed to demonstrate the functionality of our method. The manufacturing steps are shown in Fig. 1.

### 2.3. Characterization

SEM images of the nanoparticles were taken using a Quanta 3-D FEG from FEI Company, USA, equipped with an EDX system. The particle size distribution was obtained using ImageJ particle analysis. XRD was done using a multipurpose Malvern Panalytical Empyrean X-rays diffractometer, and FTIR was performed using a PerkinElmer Spectrum 100 FTIR spectrometer. X-ray photoelectron spectroscopy (XPS) analysis was carried out using an Escalab 250XI spectrometer (ThermoFisher Scientific, UK). The thickness of the copper lines was measured using a KLA-Tencor P-7 profilometer, and their resistivity using a 2-point probe technique. SEM images of the Cu tracks were taken using a Pioneer Raith microscope. To test the functionality of the 3D circuits, a LED was connected and illuminated with a 3 V power source, and a conventional perovskite solar cell (MAPbI-Cl_2_) was assembled and tested using the ABET Technologies Sun 2000 solar simulator. The power was measured using a TCS – RO3 I–V tracer.

## 3. Results and discussions

### 3.1. Silver nanoparticle formation and FTIR analysis

**Fig. 2:**
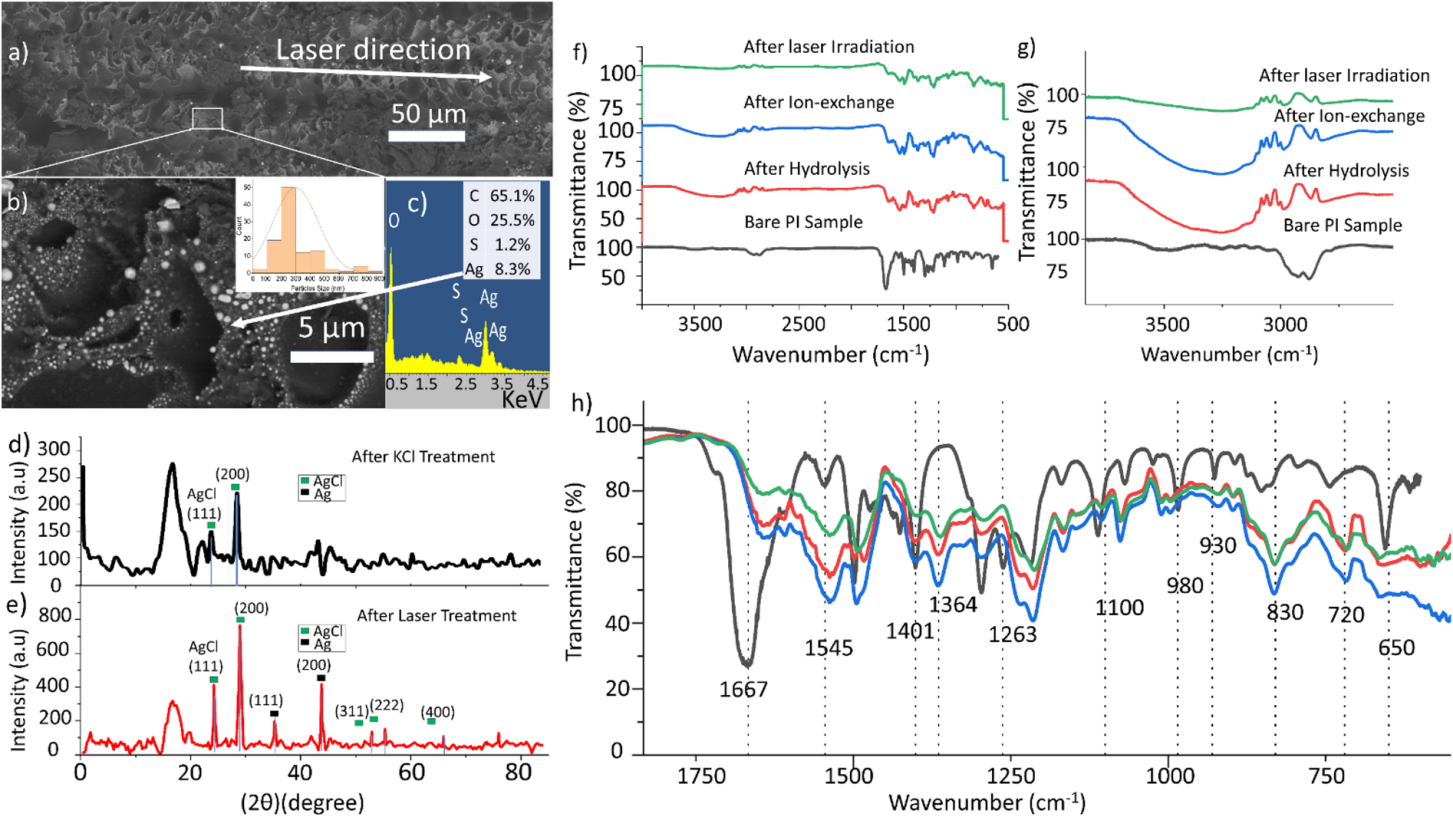
(a) SEM of a sample after laser exposure. (b) Silver nanoparticles with size dispersion. (c) EDX taken on top of a nanoparticle. (d) XRD before laser exposure. (e) XRD after laser exposure. (f) FTIR analysis for PI samples after hydrolysis, after AgNO_3_ immersion and LED exposure. (g) OH stretch after the hydrolysis process and CH_2_ asymmetric stretches. (h) Change of the PI structure after chemical treatment and laser exposure.

Fig. 2a shows a treated PI sample after laser writing in a closed-packed scanning pattern. It is possible to observe bright particles defining the tracks patterned by the laser in horizontal direction. A closer SEM inspection (Fig. 2b) reveals that the bright particles are mostly round with large dispersion size, in the range from 77 nm up to 844 nm diameter, with average size 297 nm. Energy dispersion X-rays analysis (EDX) on top of a large particle results in a percentage of atomic composition (Fig. 2c) where silver is well represented (8.3% of atomic composition, and 42.1% in weight) and sulphur is also present (1.2% atomic). The origin of the sulphur could be in the original PI resin or may be due to the cleaning step after laser irradiation, which uses immersion in a 5% sulphuric acid solution, as explained in the Experimental section. The C (65.07%) and O (25.5%) results from the polymer background, since in EDX, the signal is recorded for a region of approximately 1 μm^2^ region. The region has been scanned by X-ray diffraction (XRD) before and after laser exposure (Fig. 2d and e, respectively). Before the laser irradiation and just after the KCl treatment, it is possible to find AgCl as the only crystalline compound. Both the (111) and the (200) planes are clearly identified. After the laser exposure, new peaks from crystalline silver appear, namely those corresponding to the (111) and (200) planes. AgCl is also present in this sample. This could be attributed to the fact that the lines of the laser patterning are not close enough to each other, which could result in AgCl remaining between them.

In order to elucidate more information about the chemical reactions undergone on the substrate, infrared spectroscopy has been used. Polyimide is insoluble in most solvents; hence, non-resinous polyimide liquid is usually supplied as a solution of polyamic acid (PAA) dissolved in NMP^27–31^ When subjected to thermal imidization, *i.e.* heating the sample to high temperatures (>200 °C), polyimide is formed.^30^ Baking at lower temperatures can result in the formation of intermediate molecular structures such as polyamide-imide films.^31^ In this work, PAA-based PI liquid was spin-coated on a 3D-printed polyetherimide (PEI) substrate and heated in an oven between 120 and 200 °C for 20 minutes. Imide formation is distinguished by the appearance of the following peaks on an FTIR spectrum: C=O stretching at 1780 and 1720 cm^−1^ (asymmetric and symmetric stretching, respectively) and C–N–C axial stretching at ∼1380 cm^−1^.^32–34^ Amides, on the other hand, are recognised by a peak between 1650–1690 cm^−1^ corresponding to C=O stretching and a peak at 1550 cm^−1^ from the C–N–H vibrations.^30,32,34^ C=O vibrations from carboxylic acids also appear at around 1680–1700 cm^−1^. Other imide peaks include the 1110 cm^−1^ C–N–C transverse stretching and C–N–C out-of-plane bending between 720–740 cm^−1^.^33^ However, these peaks are very close to the alkoxy/phenyl C–O stretch at 1105 cm^−1^ and the C=O bending vibrations at 720 cm^−1^, hence these peaks are difficult to be unambiguously identified.^30,34^

In the bare PI sample in Fig. 2f, the characteristic doublet of imide C=O is absent. The prominent peak at 1670 cm^−1^ is unusually broad and encompasses smaller adjacent peaks, which indicates the presence of C=O groups from secondary amides as well as acid groups. A minor peak at 1550 cm^−1^ indicates vibrations from the C–N–H groups, while the peak at 1400 cm^−1^, although close to the characteristic C–N–C stretching, could also be also linked to phenolic C–O and O–H interaction.^33^ Similarly, the peaks at 1110 and 740 cm^−1^ could belong to either C–N–C or C–O/C=O vibrations as mentioned earlier. In either case, the above analysis points towards an incomplete cyclization (closure) of the imide rings, which is not unusual for partially cured PAA-derived PI films.^27,31^ To our benefit, it is the presence of these open rings which make PAA/PI film sensitive to chemical modifications. A few other notable peaks are: 1500 cm^−1^ (C–C stretching from phenylene group), doublet ∼2900 cm^−1^ (saturated C–H stretching), the near-absent N–H stretching at ∼3500 cm^−1^ and so on.^27,32,34,35^ Following hydrolysis of our PI samples using KOH, the C=O peak at 1670 cm^−1^ disappears, leaving behind the 1645 cm^−1^ C=O peak. The 1400, 1110 and 740 cm^−1^ peaks (likely to be C–O vibrations) become insignificant while prominent peaks appear at 1360 cm^−1^ (C–N–C) and 720 cm^−1^ (C=O bending) (a negligible peak is also seen at 1105 cm^−1^). Interestingly, the 1545 cm^−1^ C–N–H vibrations also become more significant after KOH treatment. It is known that, during hydrolysis, the imide rings are cleaved into O=C–O^−^ and O=C–N–H groups and since the imide rings in PAA only underwent partial imidization, it is highly likely that any positive metal ion such as K^+^ (and Ag^+^) bonds with the O- of the acid groups.^19^ A broad hydroxyl peak (OH) also appears in the 3700–3100 cm^−1^ region (Fig. 2g), which further confirms the formation of hydroxyl end groups due to the cleavage of the imide chains.^36^ Further steps in our treatment process do not alter the functional group distribution (Fig. 2h); the only difference seen is the change in the relative intensities of the 1545 and 1500 cm^−1^ peaks – increasing initially during the hydrolysis and reducing during the Ag^+^ exchange (with K^+^) and subsequent laser-induced Ag nanoparticle formation.

### 3.2. XPS

**Fig. 3:**
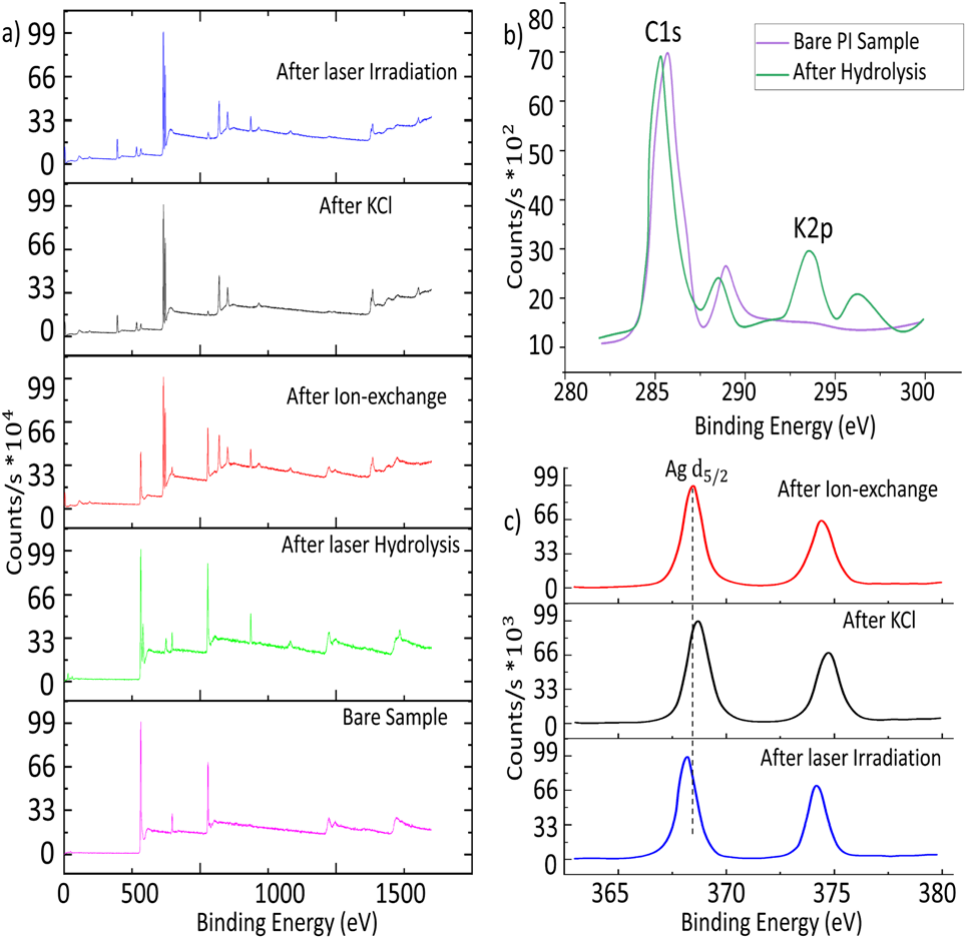
(a) XPS survey scans for PI taken after each step. (b) C 1s scan for the PL bare sample & after hydrolysis. (c) Ag 3d narrow scan for PI during sensitization and after laser irradiation.

XPS was carried out to analyse the surface chemistry of the coated samples. XPS survey scans of the samples after each step are presented in Fig. 3a. The most remarkable feature is the appearance of peaks at around 370 eV after the ion exchange, which are due to the effective presence of silver. The XPS narrow scan in the 280–300 eV region reveals the appearance of the potassium K 2p peak at 294 eV, revealing the effective incorporation of the potassium, near the carbon C 1s peak (at 284.6 eV), as shown in Fig. 3b. Fig. 3c presents two silver peaks corresponding to Ag^0^ and Ag^+^. Several positions of the sample were measured in order to obtain as accurate values as possible because identifying the causes of their energy shifts is difficult. The Ag 3d_5/2_ peak is located at 369.09 eV for PI after ion-exchange, at 368.46 eV after the KCl treatment, and at 368.25 eV after laser radiation. For the sample after laser irradiation, the peaks have values with lower binging energy. However, metallic silver is reported to have higher binding energy than AgCl,^37,38^ then the opposite trend would be expected. This could point to the possibility that the metal surface of the Ag nanoparticles is oxidized.^39^ Despite this possible oxidation, the compound still acts as a seeding layer for the electroless plating, as will be shown in the next section.

### 3.3. Laser photo-patterning and copper track deposition

**Fig. 4:**
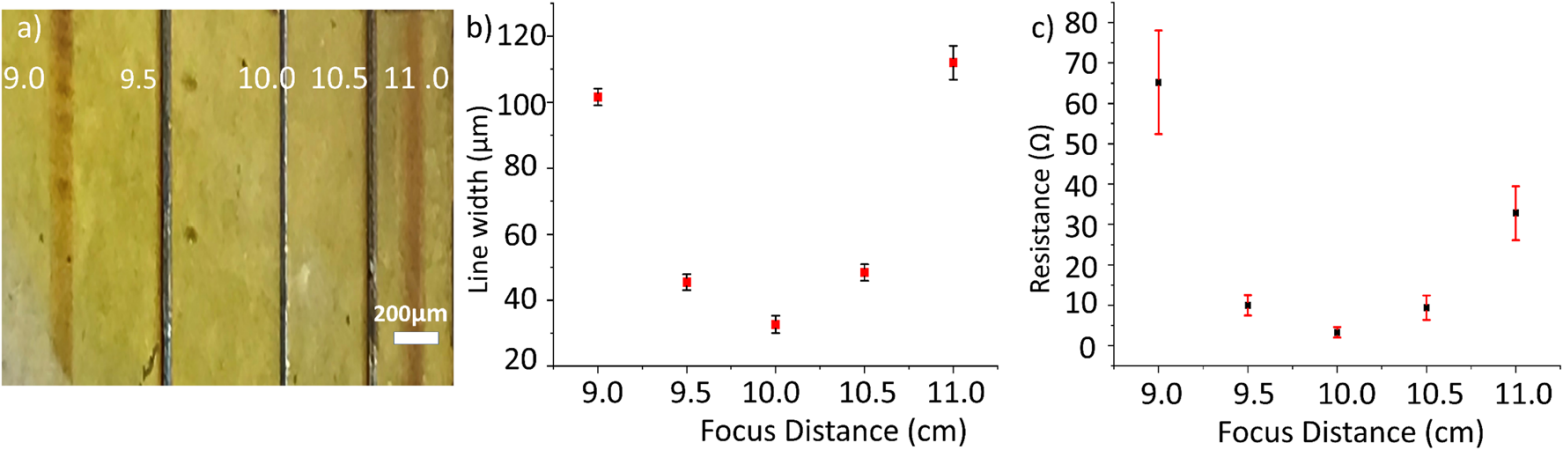
(a) Copper lines patterned at different sample-to-laser distances. (b) Graph of the correlation between distance and track width. (c) Graph of the track resistance and the patterning distance.

A low-cost diode laser is used for photo-patterning. The power of the laser when focussed on the PI sample is 32 mW. Rastering the focussed laser beam programmatically over the substrate surface produces visible lines with widths of 28 ± 5 μm when in focus. It is known that Ag nanoparticles show good absorption peak in the blue wavelength attributable to surface plasmon resonance. This enables the use of a blue laser as a heat source to selectively induce Ag NP formation from AgCl reduction. The depth of focus effect was checked by adjusting the distance between the sample and the laser source. Fig. 4a shows an optical microscope image demonstrating the varying copper track thickness when patterning at different distances from the substrate to the laser with the same laser power. Measured optically, the width of these lines is plotted against the sample-to-laser distance in Fig. 4b. Patterning at 9 and 11 cm resulted in wider lines with widths of approximately 102 and 112 μm respectively. When the distance is changed to 9.5 and 10.5 cm, the line widths decrease to approximately 46 and 48 μm; the line colour changes to dark brown, indicating an increase in the light intensity closer to the focusing point. The best and lowest width of about 32 μm is obtained at 10 cm; the line also assumes a grey colour, indicating that the focal point has been reached. The intensification of the line colour suggests that the increase of irradiation augments the reduction of the silver. This could be translated subsequently into a higher conductivity of the plated copper, because thicker films that grow on an efficient seed-layer are of higher quality.^5^Fig. 4c shows experimental results of the effect of varying the laser-to-sample distance on the resistance of the deposited Cu lines. The laser used in this work has a focused beam with the focal point (the smallest diameter of the laser spot) located at 10 cm. Therefore, patterning at different distances results in different patterned lines. The lowest resistance for a 10 mm track is found when the sample is at the focal point (10 cm), with a value of 3.3 ± 1.3 Ω, and conductivity of (1.2 ± 0.7) × 10^7^ S m^−1^, which is 20% that of pure bulk copper. If the distance is varied by 5 mm, the resistance increases to 10 ± 2.5 Ω (at 9.5 cm) or to 9.4 ± 3.0 Ω (at 10.5 cm). If the distance is 10 mm out of focus, the resistance increases to 65 ± 13 Ω (at 9.0 cm) or to 32.8 ± 6.6 Ω (at 11.0 cm). The variations of the resistance is not only due to the different width of the lines, but also to the quality and speed of the plating, which depends on the seed layer quality. From this experiment it is deduced that the laser writing technique has some depth of focus, which provides tolerance to the technique, because it is not necessary to have the sample always at the focus, although the resistance increases when out of focus.

**Fig. 5:**
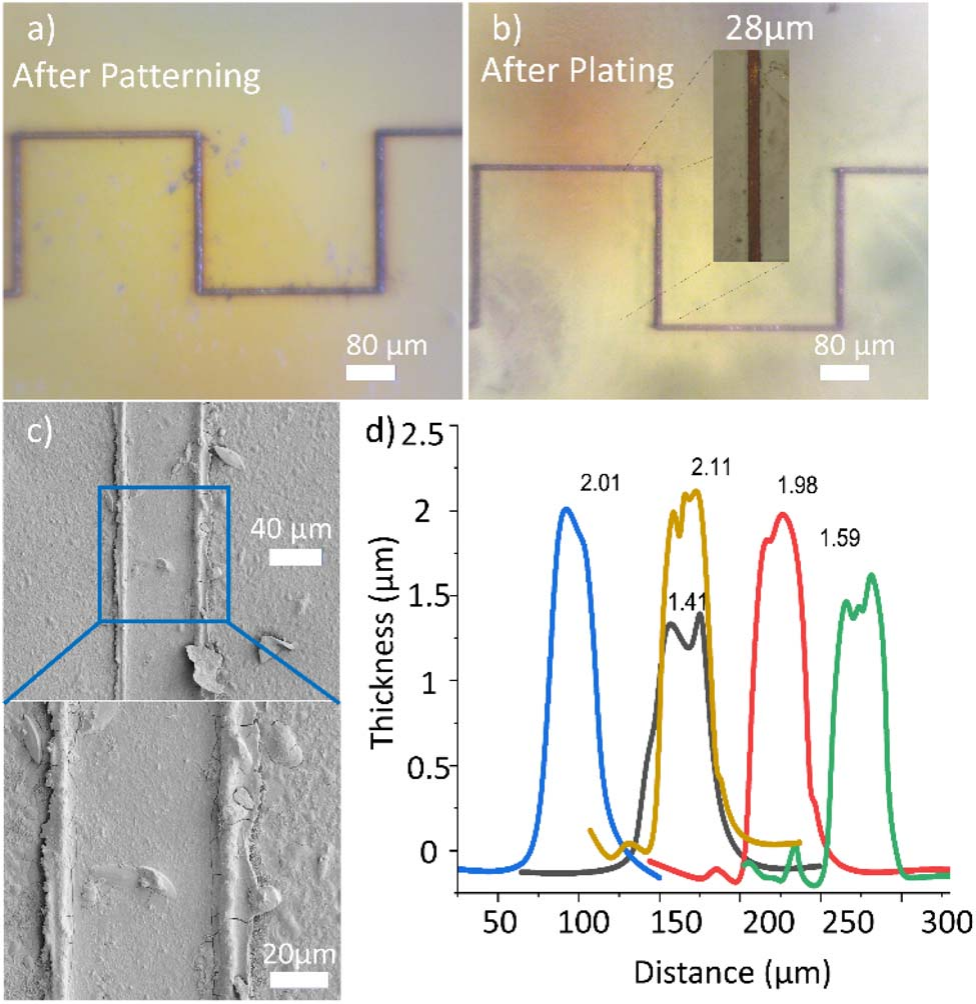
The laser patterning in the substrate. (a) PI coated sample just after laser patterning, (b) sample after Cu plating of the square shape. (c) SEM scans for deposited copper lines. (d) Profilometric scan demonstrating the thickness of copper line.

Fig. 5 shows samples just after the laser writing, and after copper is deposited by electroless-plating at 50 °C for 5 minutes. The samples were produced using photopolymer resin for 3D printing and coated with PI. Fig. 5a shows a simple 2D pattern with a square wave shape just after the laser writing, and Fig. 5b is the sample after electroless copper plating. The inset is a zoomed image of the plated line showing its measured width. It can be seen that copper tracks with widths similar to those of the underlying seed-layer tracks are formed, thus confirming the excellent selective deposition of copper. The average copper thickness of these lines is 1.8 ± 0.3 μm and the average electrical conductivity is (1.2 ± 0.2) × 10^7^ S m^−1^. To investigate the surface morphology of the deposited copper, the samples were scanned with SEM. In Fig. 5c, it can be observed that the copper morphology is uniform and smoother than the unplated regions of the sample. The edges of the copper track are swollen compared to the central region, which indicates partial melting by laser irradiation. The edges of the ridge show some cracks, but there are no cracks on the central region of the copper line. Fig. 5d is a profilometric plot showing the thickness of the deposited copper at different areas along the line. The measurements showed that the thickness varies between 1.41 and 2.11 μm, giving an average thickness of 1.8 ± 0.3 μm.

### 3.4. Circuitry on 3D geometry

**Fig. 6:**
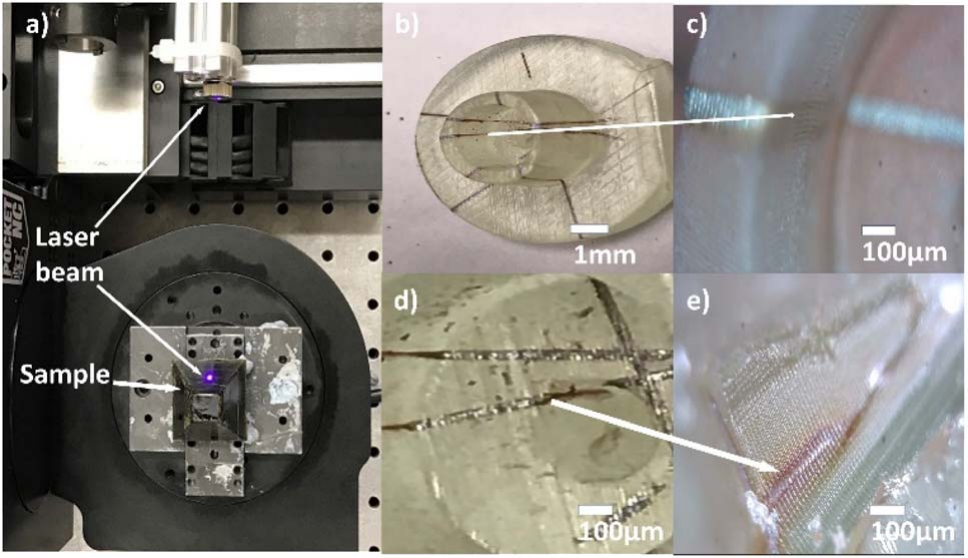
(a) 5-axis machine while patterning, (b–e) copper-plated inside pores and on 3D-printed geometry to fabricate micro 3D electric circuit.

The direct laser writing method can also be used to write 3D circuits as depicted in the photographs in Fig. 6. Fig. 6a is a photograph of the laser writing which consists of a laser attached to a 5-axis CNC machine; the laser beam is directed towards the stage which holds the 3D printed geometry. Fig. 6b shows the sample after laser writing. Clear lines of high reflectivity can be seen inside the pores, confirming that Ag^0^ has been selectively formed in the inner surface inside the hole.

The path of copper tracks on the 3D-printed geometry starts from the base to the top of the hollow cylindrical projection Fig. 6b and down to the base on the inner side, showing selective and uniform deposition of copper. Fig. 6c is an image of the 3D geometry after copper electroless plating which shows distinct copper tracks. The average width of the copper lines is 61 ± 10 μm at the wider area of the line, which represents off-focal point, and about 30 ± 2 μm when perfectly focussed. Given that the laser focus is not adjusted during the writing process, small differences in widths are to be expected on inner or curved surfaces. The copper track on the top is well formed, whereas in areas such as that shown in Fig. 6d and e, the copper lines are incomplete. The shape of the geometry will be important given that, to get the photopatterning, it is required that the laser can reach the surface. Complicated geometries, or closed cavities, can prevent the laser of reaching the surface. This happens in Fig. 6d and e where the complicated geometry (a hole inside a larger cavity) prevents the writing of the most inner region due to shadowing. Another challenge in using this setup is the fact that this diode laser produces an elliptical beam which results in differences in width between horizontal and vertical lines. Finally, the spin coating step will be only applicable for planar or simple 3D geometries, while other deposition methods such as immersion or spray coating could extend the range to more complicated geometries.

**Fig. 7:**
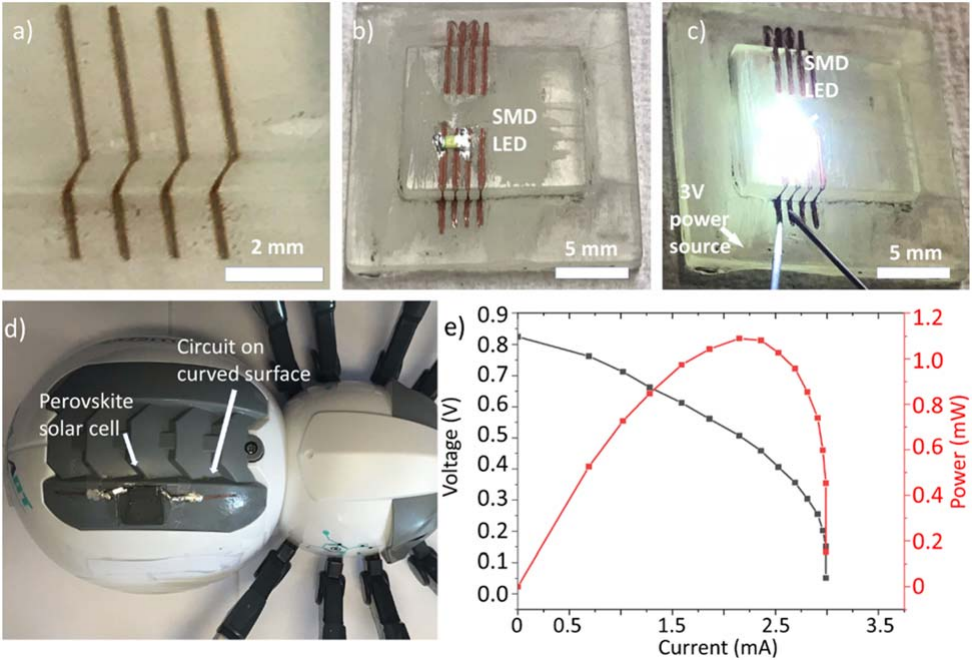
(a) 3D copper lines on 3D printed acrylate, patterned and plated using laser writing. (b) Soldered SMD LED using silver paste. (c) Functional LED using 3D circuits. (d) Circuit manufactured on a curved ABS surface for solar cell integration. (e) *I*–*V* curve and power output of the used perovskite solar cell.

In order to demonstrate the performance of this technique, a circuit on a 3D-printed substrate has been manufactured. The substrate has a 90-degree step, which would prevent the use of other interconnecting techniques. After processing, the sample is plated for 5 min., and clear copper tracks are shown on the sample Fig. 7a. A surface mount device (SMD) LED is soldered using silver paste on a sample written with higher defocussing, which provides thicker lines, as shown in Fig. 7b. A 3 V DC source is then connected Fig. 7c. When the source powers the circuit, the LED switches on, which indicates a fully functional 3D-printed electrical circuit. The writing process could be improved by adjusting the code in order to track the surface and keep the focal point on the interface, which would achieve the highest possible resolution.

As proof-of-concept, a basic electrical circuit was prepared on a commercial robot, as seen in Fig. 7d. PI was applied by blade coating and baked at 70 °C for 1 hour in a Carbolite oven. The electrical circuit was patterned on the ABS curved surface using the 5-axis machine and direct laser writing. A perovskite solar cell with dimensions of 1.5 × 1.5 cm^2^ was attached to the circuit using adhesive and soft soldering using silver paste as described in ref. 25. One of the advantages of this solar cell technology is that it can be deposited on flexible and curved geometries. For this reason, this technology can benefit from an integration method as presented in this work. Fig. 7e shows the cell performance measured on the solar simulator under the AM 1.5 solar spectrum. The *I*–*V* curve demonstrates that the open circuit voltage (*V*_oc_) starts at 0.82 V, and the maximum short circuit current (*I*_sc_) is 2.9 mA, with maximum power (*P*_max_) of approximately 1 mW, which are normal values for this kind of experimental solar cell.

## 4. Conclusions

This paper demonstrates the use of an inexpensive direct laser writing method to produce 2D and 3D electronic circuits on various substrates. Liquid polyimide-coated 2D and 3D substrates are chemically sensitised and exposed to focussed laser beam to produce tracks of silver nanoparticles. FTIR and XPS monitoring of the surface chemical changes confirm imide cleavage and bonding of Ag nanoparticles to the substrates. As a second step, copper is deposited on silver tracks by electroless-plating. Optical micrographs confirmed well-defined copper tracks. A track width of 28 μm with an average copper thickness of 1.8 ± 0.3 μm and an electrical conductivity of (1.2 ± 0.2) × 10^7^ S m^−1^ is achieved, which proves that the technique is a suitable selective metallization technique for depositing copper on complex geometry. The technique has shown to have some tolerance to variations on the sample-to-laser distance, which makes easier the patterning because the sample doesn't have to be closely tracked. Proof-of-concept 3D circuits were fabricated, and their working demonstrated. Finally, the method was used to integrate a perovskite solar cell on an ABS commercial robot.

## Conflicts of interest

There are no conflicts to declare.

## Supplementary Material

NA-005-D3NA00120B-s001
