# Supplementary material for: Low-power laser manufacturing of copper tracks on 3D printed geometry using liquid polyimide coating
Source: Nanoscale Adv. 2023 Mar 18;5(8):2280–7. doi: 10.1039/d3na00120b (PMC10089081; doi:10.1039/d3na00120b)
Supplement: NA-005-D3NA00120B-s001 [file NA-005-D3NA00120B-s001.pdf]

Electronic Supplementary Material (ESI) for Nanoscale Advances.
This journal is © The Royal Society of Chemistry 2023

Electronic supplementary information (ESI) – Nanoscale Advances, 2023

Low-power laser manufacturing of copper tracks on 3D printed geometry using liquid polyimide coating

Mansour Abdulrhman[a], Adarsh Kaniyoor[a], Carmen M. Fernández-Posada[b], Pablo Acosta-Mora[a], Ian McLean[c], Nick Weston[c], Marc. P. Y. Desmulliez[a], Jose Marques-Hueso*[a]

The adhesion of the polyimide film (PI-1388 Liquid Polyimide, from VTEC™) has been tested by using the scotch tape test, which refers to the Standard IPC-TM-650 scotch-tape test (3 M 600 scotch tape). As expected, the PI presents a good adhesion to the tested 3D printing materials (ABS, PLA and acrylic photopolymer), and it is expected to work with any other material that offers enough adhesion. The tape didn't pull any pieces of the PI film, and it is only observed that the glue of the tape is roughened.

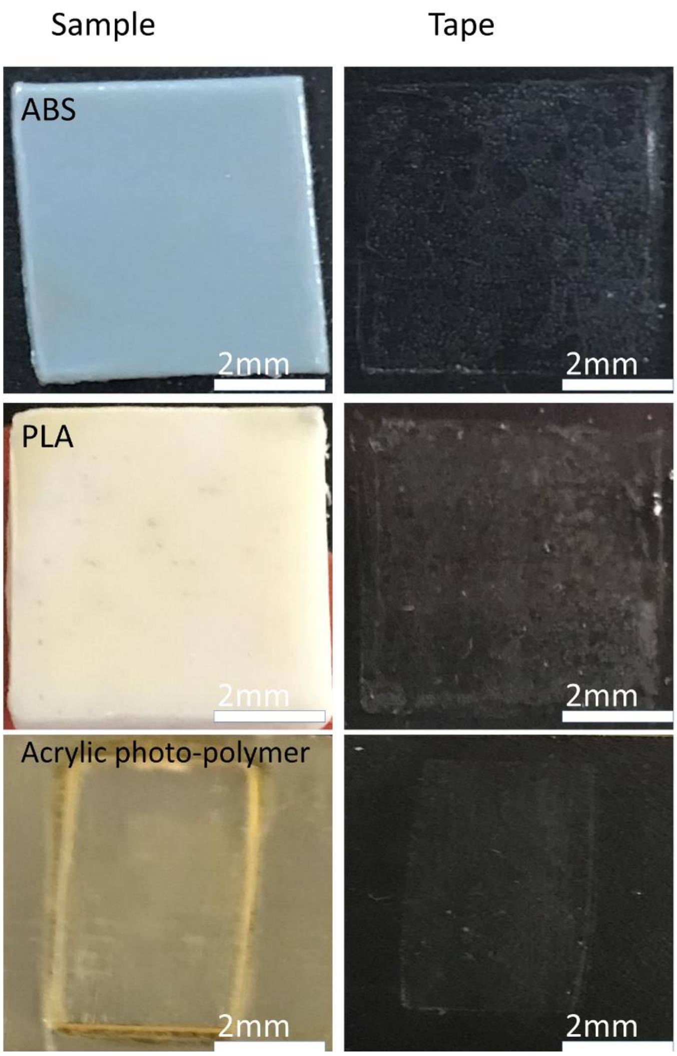

Fig. S.1. ABS, PLA and Acrylic samples coated with PI, and the corresponding scotch tape after contacting the sample and being pulled. No pieces of PI have been peeled.
